# Supplementary material for: Arabidopsis Hypocotyl Adventitious Root Formation Is Suppressed by ABA Signaling
Source: Genes (Basel). 2021 Jul 27;12(8):1141. doi: 10.3390/genes12081141 (PMC8392626; doi:10.3390/genes12081141)
Supplement: Supplementary file 1 [file genes-12-01141-s001.zip › genes-1263031-supplementary.pdf]

Supplementary Materials:

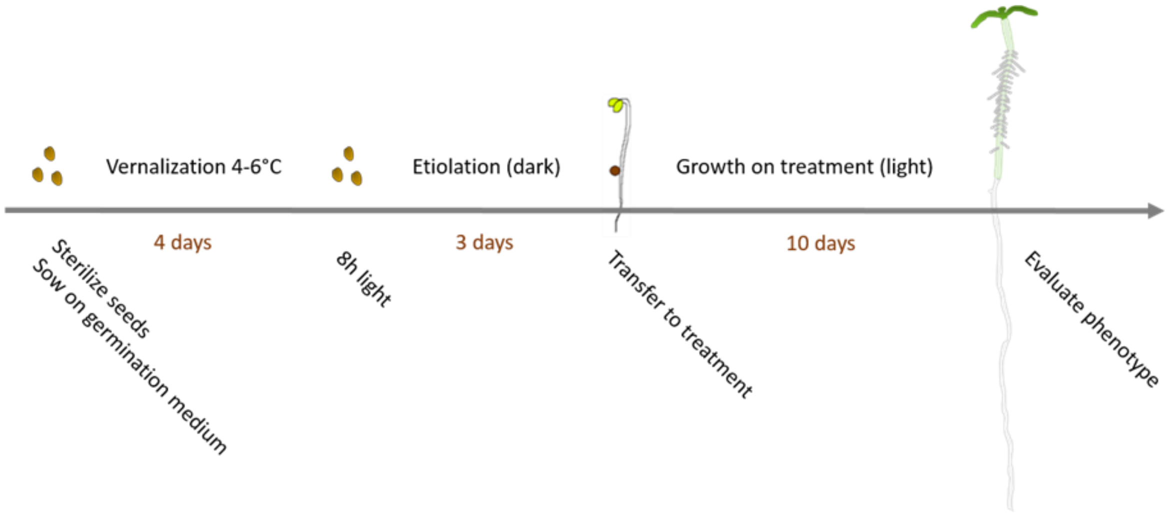

Figure S1. Growth and sampling scheme of Arabidopsis thaliana seedlings in the adventitious root assay.

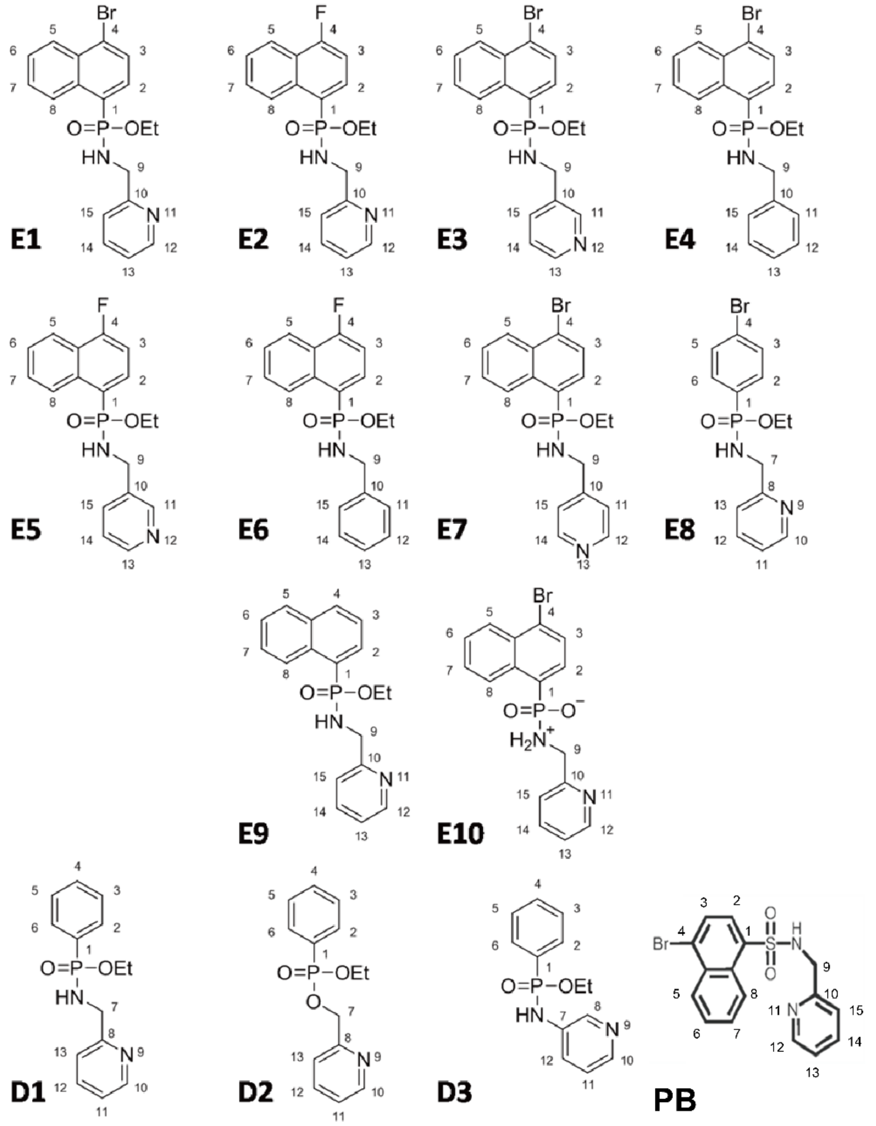

Figure S2. Chemical structures of phosphonamide (E1-E10) and phosphonate (D1-D3) pyrabactin analogues. E1, Ethyl N-(pyridin-2-ylmethyl)-P-(4-bromonapht-1-yl)phosphonamidite; E2, Ethyl N-(pyridin-2-ylmethyl)-P-(4-fluoronapht-1-yl)phosphonamidite; E3, Ethyl N-(pyridin-3-ylmethyl)-P-(4-bromonapht-1-yl)phosphonamidite; E4, Ethyl N-(benzyl)-P-(4-bromonapht-1-yl)phosphonamidite; E5, Ethyl N-(pyridin-3-ylmethyl)-P-(4-fluoronapht-1-yl)phosphonamidite; E6, Ethyl N-(benzyl)-P-(4-fluoronapht-1-yl)phosphonamidite; E7, Ethyl N-(pyridin-4-ylmethyl)-P-(4-bromonapht-1-yl)phosphonamidite; E8, Ethyl N-(pyridin-2-ylmethyl)-P-(4-bromophenyl)phosphonamidite; E9, Ethyl N-(pyridin-2-ylmethyl)-P-(napht-1-yl)phosphonamidite; E10, Hydrogen N-(pyridin-2-ylmethyl)-P-(4-bromonapht-1-yl)phosphonamidite; D1, Ethyl N-(pyridin-2-ylmethyl)-P-(phenyl)phosphonamidite; D2, Ethyl (pyridin-2-ylmethyl) phenylphosphonate; D3, Ethyl N-(pyridin-3-yl)-P-(phenyl)phosphonamidite. PB, pyrabactin.

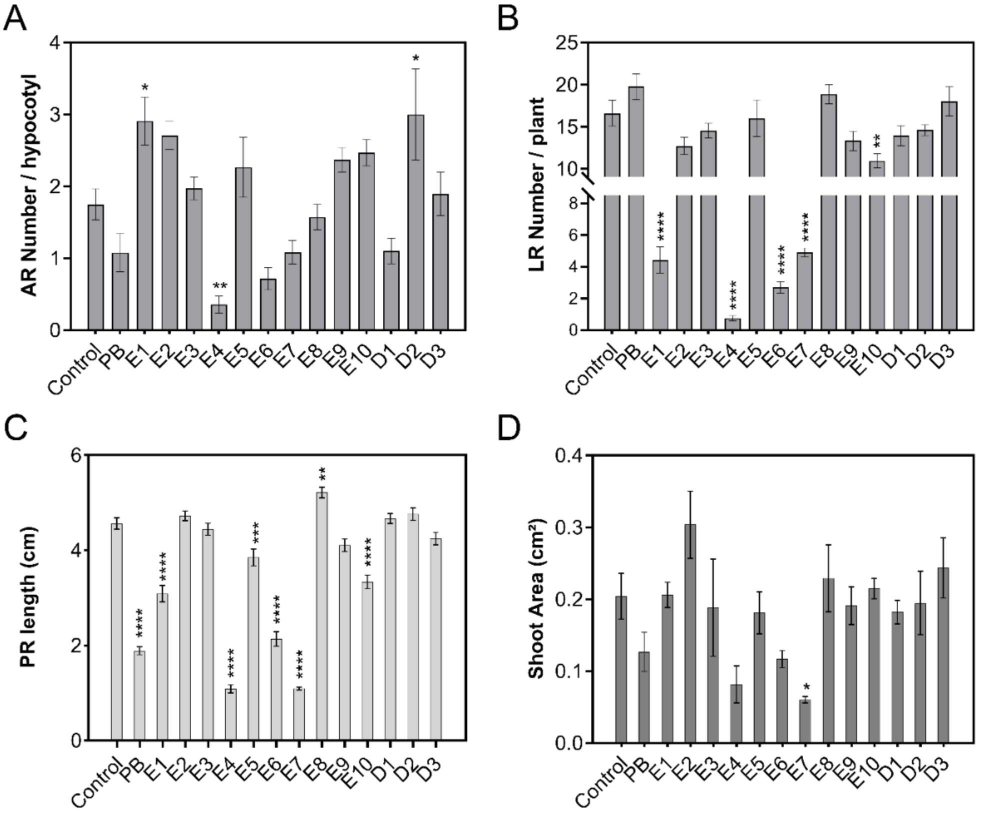

Figure S3. Phenotypic characterization of novel PB analogs. Col-0 plants were grown for 10 days after stratification following 3 days of etiolation in the dark. Quantification of the primary root growth (A), lateral root number per plant (B), adventitious root number per hypocotyl (C) and shoot area (D). Plants in the absence or presence of 10 μM PB or PB analogues. Data are represented as mean values ± se were obtained from plants grown in three independent experiments (n = 20–25). Statistics were calculated through ANOVA and levels of significance are represented as (*) P ≤ 0.05, (**) P ≤ 0.01, (***) P ≤ 0.001 and (****) P ≤ 0.0001.

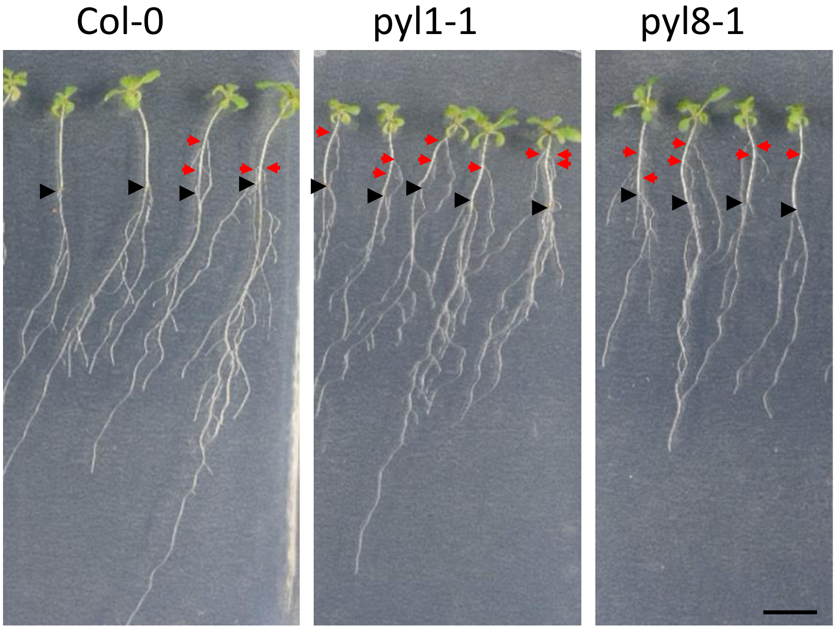

Figure S4. Phenotype of pyl1-1 and pyl8-1. Black arrow heads indicate the position of the hypocotyl root junction; red arrows indicate the origins of AR. Pyl1-1 and Pyl8-1 display a root architecture that is similar to the WT control Col-0, albeit that pyl1-1 and pyl8-1 showed a tendency to produce slightly more AR.

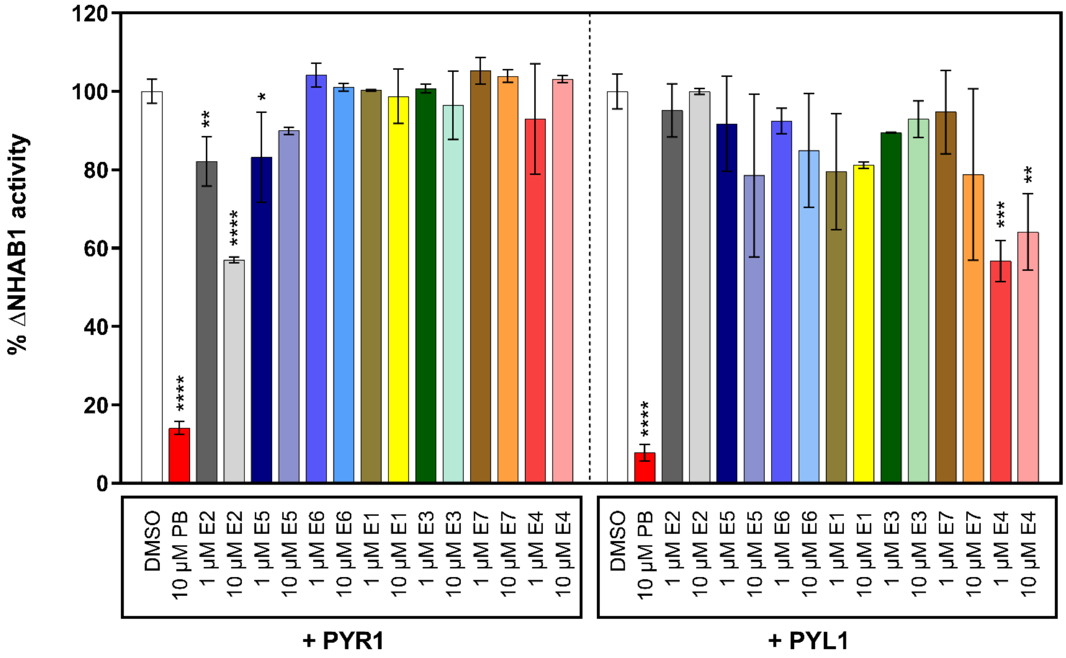

Figure S5. Effect of PB and PB analogs on PP2C phosphatase activity. Phosphatase activity was measured using pNPP as a substrate and PP2C ΔNHAB1 and either PYR1 or PYL1. PB was applied at 10μM and the PB analogs at 1 or 10μM. E2 inhibited PYR1 and E4 inhibited PYL1-mediated ΔNHAB1 phosphatase activity. Data are represented as mean values ± sd obtained from three independent experiments. Statistics were calculated through ANOVA and levels of significance are represented as (*) P ≤ 0.05, (**) P ≤ 0.01, (***) P ≤ 0.001 and (****) P ≤ 0.0001.

Table S1. List of PCR primers.

Name	Locus tag	Primer sequences (5′ - 3′)
pyr1-1_F	At4g17870	TAA AAG CTC GTC GTC GTC TTC
pyr1-1_R		GGA AAA GAA AAG GAA AAC CTT TC
pyl1-1_F	At5g46790	ATGGCGAATTCAGAGTCCTCC
pyl1-1_R		TTACCTAACCTGAGAAGAGTT
pyl2-1_F	At2g26040	ACCATGGGCTCATCCCCGGCCGTGA
pyl2-1_R		TTATTCATCATCATGCATAGGTG
pyl3 _F	At1g73000	AGG AGC AAT TTG AAC TCC CTC
pyl3_R		TTG GAA ACC TGG ATT GTT GAC
pyl4-1_F	At2g38310	ACCATGGTTGCCGTTCACCGTCCTT
pyl4-1_R		TCACAGAGACATCTTCTTCTTGC
pyl5_F	At5g05440	ATGAGGTCACCGGTGCAACT
pyl5_R		TTATTGCCGGTTGGTACTTCGA
pyl8-1 _F	At5g53160	ATGGAAGCTAACGGATTGAG
pyl8-1_R		TTAGACTCTCGATTCTGTCGT
pyl9_F	At1g01360	TTC ACT TCA ATG CCC TTG TTC
pyl9_R		TAG GTC CCC AAA ACG TCA TAC
LBb1.3		ATT TTG CCG ATT TCG GAA C

Table S2. Overview of bioactivity scoring of phosphonamide and phosphonate pyrabactin analogs. All the molecules were applied at 10 μM after etiolation and scoring was done after transfer to light for 10 days. The impact of the compounds was compared with control not-treated seeds and seedlings and scored as not different from control (0), strong action (+++), intermediate action (++), weak action (+) and opposite action (-).

Analogue	Seed germination	PR growth	LR initiation	AR initiation	Shoot development
ABA	+++	+++	+++	++	+++
PB	++	++	++	+	++
E1	0	+	++	-	0
E2	0	0	+	-	-
E3	0	0	+	-	0
E4	+	+++	+++	+++	+++
E5	+++	+	+	0	0
E6	0	++	+++	++	++
E7	0	+++	+++	+	+++
E8	0	0	+-	0	0
E9	0	0	+	-	0
E10	0	+	+	-	0
D1	+	0	+-	0	0
D2	0	0	0	-	0
D3	0	0	0	0	0
